# The Green and Effective Synthesis of Isoxazole-Based Molecules Under Ultrasonic Irradiation Approaches

**DOI:** 10.3390/ph18081179

**Published:** 2025-08-10

**Authors:** Mei-Tong Chen, Yao-Rong Li, Zhao-Qi Wang, Shan Jiang, Zan-Hui Jia, Da-Wei Zhang

**Affiliations:** 1 The Second Hospital of Jilin University, Changchun 130041, China; 18646336867@163.com (M.-T.C.); drjzqd@163.com (S.J.); 2 College of Plant Science, Jilin University, Changchun 130062, China; 3 College of Food Science and Engineering, Jilin University, Changchun 130062, China; liyr9921@mails.jlu.edu.com; 4 College of Chemistry, Chemical Engineering and Resource Utilization, Northeast Forestry University, Harbin 150040, China; 13029708008@163.com; 5 College of Chemistry, Jilin University, Changchun 130012, China

**Keywords:** isoxazole, ultrasonic irradiation, heterocyclic compound, green, synthesis

## Abstract

Isoxazole-based molecules constitute a crucial category of heterocyclic compounds with wide-ranging applications across pharmaceutical development, advanced materials, and pesticide synthesis. Traditional synthetic approaches for isoxazole derivatives frequently encounter challenges such as extended reaction periods, severe operating conditions, and reliance on toxic solvents. As an eco-friendly alternative, sonochemistry has emerged as a promising approach for organic synthesis, offering enhanced reaction efficiency, reduced energy consumption, and improved yields. In this context, this review introduces the recent advancements in ultrasound-assisted strategies for the synthesis of isoxazole-scaffolds and their derivatives. Various methodologies are discussed, including multi-component reactions, catalytic systems, and solvent-free protocols. The integration of ultrasound not only accelerates reaction kinetics but also minimizes byproduct formation and enables the use of green solvents or catalysts. Key advantages such as shorter reaction durations, higher atom economy, and operational simplicity are emphasized. This work underscores the potential of sonochemical techniques to revolutionize isoxazole-based molecule synthesis, aligning with the principles of sustainable and green chemistry.

## 1. Introduction

**Figure 1:**
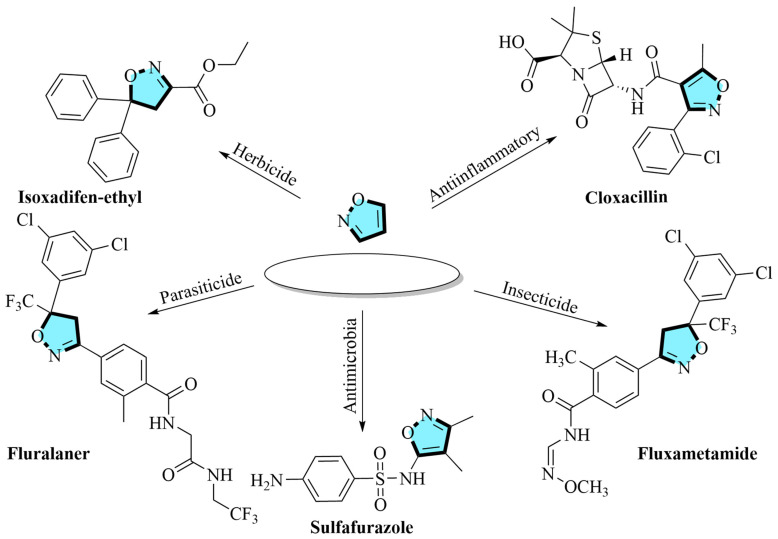
The chemical structure and application of commercialized isoxazole-containing molecules.

Isoxazole, a pair of isomers of oxazole structured as a five-membered heterocyclic ring containing one oxygen and one nitrogen atom, has emerged as an important scaffold in medicinal chemistry, materials science, and agrochemical research. The structural versatility of isoxazole derivatives enables diverse biological activities, including antimicrobial, antiviral, antioxidant, anticancer, and anti-inflammatory properties [1,2,3,4,5,6,7,8,9,10,11]. Notably, isoxazole motifs are integral to several clinically approved drugs, such as the antibiotic linezolid and the anticancer agent bleomycin. Beyond pharmaceuticals, isoxazole-based compounds find applications in fluorescent materials [12], coordination chemistry [13], and organic electronics [14], underscoring their multidisciplinary significance. Some commercialized isoxazole-containing molecules used in practical applications are shown in Figure 1 [15,16,17,18,19].

Conventional synthetic routes to isoxazole derivatives, such as the cyclization reaction [20,21,22] often suffer from limitations including harsh reaction conditions and the use of highly toxic transition-metal catalysts such as copper metals [23]. These drawbacks highlight the urgent need for green and sustainable methodologies that enhance efficiency, reduce energy consumption, and minimize environmental impact.

Ultrasound-assisted synthesis has emerged as a transformative tool in organic chemistry in recent decades [24,25]. The advantages of this method are mainly based on the acoustic cavitation induced by ultrasonic irradiation (20~100 kHz), a phenomenon where microbubbles form and collapse in the reaction medium, generating localized high temperatures (~5000 K) and pressures (~1000 atm). This mechanical energy enhances mass transfer, accelerates reaction kinetics, and promotes the formation of reactive intermediates, thereby improving yields and reducing side reactions. Crucially, sonochemistry aligns with green chemistry principles by enabling reactions under milder conditions, reducing solvent volumes, and often eliminating the need for toxic catalysts [26,27].

Ultrasound-assisted organic synthesis has many applications, and heterocyclic synthesis is one of the important and successful methods [28,29,30]. For instance, for the synthesis of isoxazole derivatives, sonochemical methods facilitate efficient cyclization, cross-coupling, and multicomponent reactions. For example, Borthakur et al. reported a method for applying an ultrasound-assisted approach to the addition reaction to generate isoxazoline and significantly reduce the reaction time—a significant improvement over traditional thermal methods [31]. These advances underscore the synergy between ultrasound and the synthesis of isoxazole derivatives in optimizing reaction pathways.

**Figure 2:**
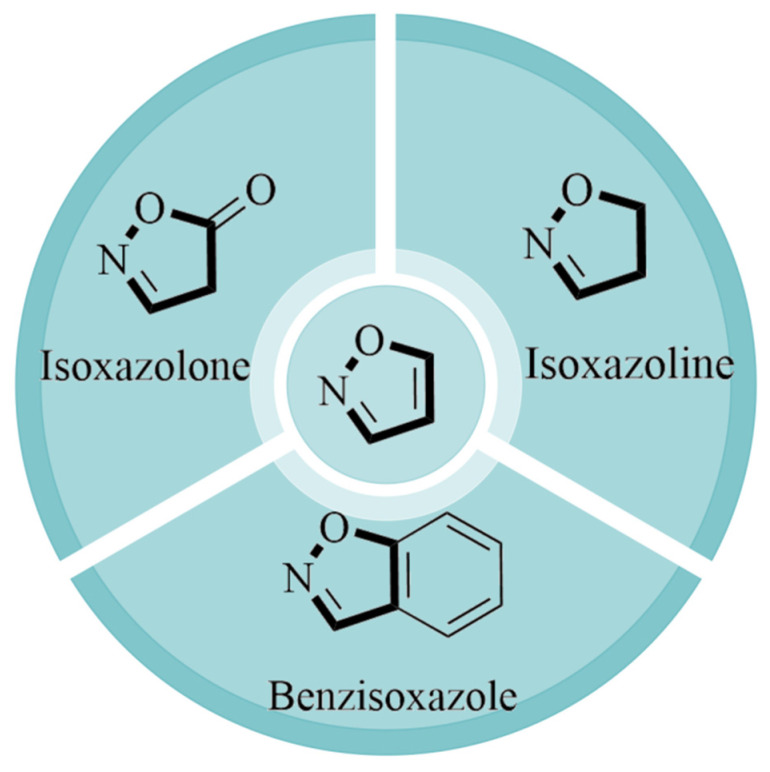
Chemical structures of isoxazole-based derivatives.

In this paper, we give an overview of the last thirty years of ultrasound-assisted organic synthesis of isoxazole-based molecules according to the differences in molecular structure of their products (Figure 2). This review systematically explores the ultrasound-assisted synthesis of isoxazole derivatives, emphasizing the mechanistic role of acoustic cavitation in enhancing selectivity and sustainability. By comparing conventional and sonochemical approaches, we elucidate how ultrasound optimizes reaction pathways, reduces energy consumption, and broadens substrate compatibility. Furthermore, we discuss emerging trends, including the use of recyclable catalysts and solvent-free systems, to inspire future innovations in green heterocyclic chemistry.

## 2. Development of Ultrasound Irradiation Synthesis of Isoxazole-Based Compounds

### 2.1. Synthesis of Isoxazole-Based Derivatives

Isoxazole skeleton, versatile N-O heterocycles. Medicinally, they serve as scaffolds for antimicrobial/antitubercular agents, Cyclooxygenase-2 (COX-2) inhibitors, anticancer compounds, and central nervous system (CNS) drugs [32]. Significantly, this skeleton is one of the top 35 most frequently occurring nitrogen heterocyclic compounds in U.S. FDA-approved drugs (January 2013–December 2023) [33].

**Scheme 1:**
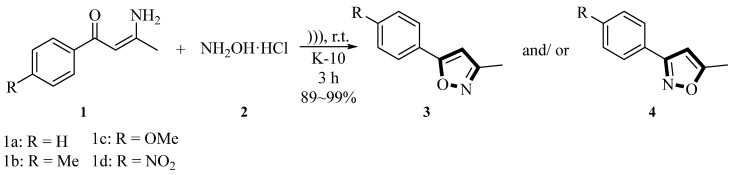
Synthesis of **3** and **4**.

In 1999, the Valduga group reported a method in which a series of β-enamino ketones **1** underwent cyclization reactions with hydroxylamine hydrochloride **2** under mild conditions, catalyzed by montmorillonite K-10 (MMT-K10) and ultrasound irradiation (50~60 Hz, 110/220 V, 1.0 A) in dichloromethane for 3 h at room temperature, to generate 3-methyl-5-phenylisoxazole derivatives **3** and/or 5-methyl-3-phenylisoxazole derivatives **4**. The yields of the isoxazole products varied depending on the substrate, ranging from 89% to 99%. Notably, **1a** and **1b** exclusively afforded **3a** (91%) and **3b** (94%), while **1c** gave a mixture of **3c** and **4c** (ratio 1.1:1.0, total yield 89%), and **1d** yielded exclusively **4d** (99%). The regiochemistry was confirmed through reductive cleavage of the N-O bond using [Mo(CO)_6_], regenerating the corresponding β-enamino ketones (Scheme 1) [34].

**Scheme 2:**
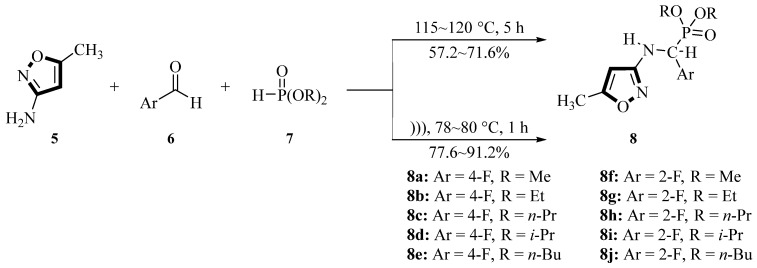
Synthesis of **8**.

In 2005, the Song group successfully utilized 5-methylisoxazol-3-amine **5**, 2-fluorobenzaldehyde or 4-fluorobenzaldehyde **6**, and dialkyl phosphite **7** to synthesize a series of 1-[(5-methylisoxazol-3-yl)amino]-1-(2- or 4-fluorophenyl)methanephosphonate derivatives **8** under ultrasound irradiation. This reaction was based on a one-pot Mannich-type reaction conducted without solvent or catalyst. Ultrasonic irradiation (25 kHz, 500 W) significantly accelerated the reaction and improved the yield, increasing from 57.2~71.6% under traditional heating (115~120 °C, 5 h) to 77.6~91.2% under ultrasound conditions (78~80 °C, 1 h). They found the position of the fluorine substituent (2- or 4-) and the alkyl chain length influenced the yield. For the same fluorine substituent, the yield order was ortho > para. These compounds exhibited moderate anticancer activity against PC3 (prostate cancer) and A431 (epidermoid carcinoma) cells in MTT assays. Notably, the compounds **8a**~**8e** showed higher inhibition rates compared with **8f**~**8j**, with compound **8a** demonstrating the strongest activity (78.3% and 69.0% inhibition of PC3 and A431 cells, respectively, at 10 µM). This synthesis method was simple, rapid, and high yielding, and marked the first report of anticancer bioactivity for these compounds (Scheme 2) [35].

**Scheme 3:**
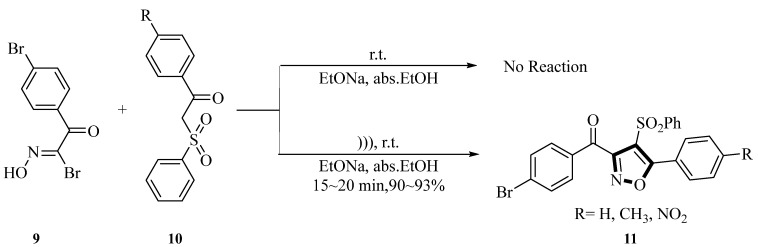
Synthesis of **11**.

In 2009, Saleh et al. [36] developed a method for synthesizing isoxazole derivatives **11** using 1-(4-bromophenyl)-2-bromo-2-hydroximinoethanone **9** and β-ketosulphone derivatives **10** under ultrasonic irradiation and with sodium ethoxide in absolute ethanol solution as a catalyst. The sodium ethoxide in ethanol solution acted as both a catalyst and a base in these reactions, facilitating the reaction by generating carbanion intermediates, neutralizing acidic by-products, and maintaining the alkaline environment of the reaction system. This step was crucial for the smooth progression and high yield of the reaction. The reaction was carried out at room temperature for approximately 15~20 min, with yields under ultrasound conditions ranging between 90% and 93% and the reaction did not proceed without sonication under the same conditions. The ultrasound-promoted reaction proved to be an effective method for synthesizing substituted isoxazoles containing sulfonyl groups. This approach not only reduced the reaction time but also improved the yield. The synthesized products exhibited high purity and did not require further purification (Scheme 3).

**Scheme 4:**
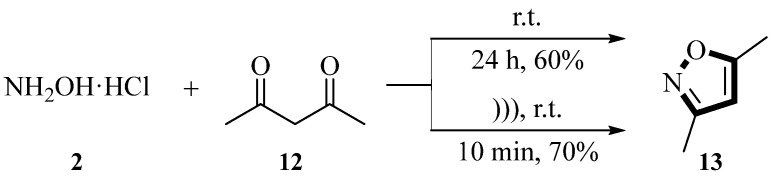
Synthesis of **13**.

In 2009, the Silva group described a reaction for synthesizing 3,5-dimethylisoxazole **13** using 2,4-pentanedione **12** and hydroxylamine **2** under ultrasound conditions. Such reactions usually yielded only intermediates, but this process yielded the isoxazole product directly in one pot. This reaction was carried out under ultrasound irradiation for 10 min in aqueous media at room temperature, without the need for catalysts, acids, or a prolonged reaction time. They also conducted antioxidant activity tests on the product. The results showed that 3,5-dimethylisoxazole **13** did not exhibit significant antioxidant activity in either the oxygen radical absorbance capacity (ORAC) or 2,2-diphenylpicrylhydrazyl (DPPH) testing methods. This method not only shortened the reaction time (from 24 h under conventional conditions to 10 min) but also improved the yield with limited increase (from 60% to 70%), providing a new and efficient approach for the synthesis of such heterocyclic derivatives (Scheme 4) [37].

**Scheme 5:**
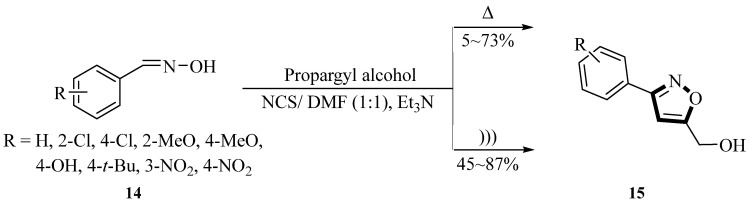
Synthesis of **15**.

In 2011, the Shen group reported the synthesis of (3-phenylisoxazol-5-yl) methanol derivatives **15** using aromatic aldehydes oxime **14**, *N*-chlorosuccinimide (NCS), dimethylformamide (DMF), triethylamine (Et_3_N), and propargyl alcohol under ultrasound irradiation. The reaction was carried out under ultrasonic conditions (250 W, 25 kHz) in one pot. Compounds bearing electron-donating groups (e.g., methoxybenzaldehyde oxime, hydroxybenzaldehyde oxime, tert-butylbenzaldehyde oxime) gave higher yields than those with electron-withdrawing groups (e.g., chlorobenzaldehyde oxime, nitrobenzaldehyde oxime). For example, when the substituent was a nitro group, the yield was only 5~7% with conventional methods, increasing to 45~59% with ultrasound-assisted methods. In contrast, when the substituent was an electron-donating group, the yield exceeded 70% under ultrasound conditions, especially when the hydroxyl group, which has strong electron-donating properties, was present, resulting in the highest product yield (87%). The yield of various (3-phenylisoxazol-5-yl)methanol derivatives **15** synthesized by the one-pot method under ultrasound irradiation ranged from 45% to 87%, with a reaction time of 90~270 min. Ultrasonic irradiation significantly accelerated the entire process, shortened the reaction time by over an hour, and increased the product yield by 14~40% (Scheme 5) [38].

**Scheme 6:**
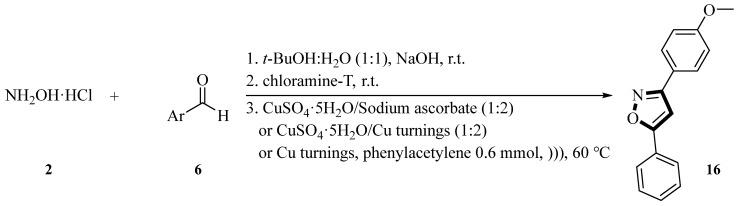
Synthesis of **16**.

In 2014, the Koufaki group synthesized 3,5-disubstituted isoxazoles **16** using hydroxylamine hydrochloride **2**, 4-methoxybenzaldehyde **6**, chloramine-T, terminal alkyne, copper sulfate pentahydrate, and sodium ascorbate or copper turnings under ultrasound irradiation. The researchers employed a one-pot, three-step process based on a copper(I)-catalyzed cycloaddition reaction, where in situ-generated nitrile oxides (from corresponding aldehydes) react with alkynes. They used *t*-BuOH/H_2_O (1:1) at 60 °C for 60 min, and the ultrasound frequency was 40 kHz (ultrasound bath) and 20 kHz (probe). The experimental results indicated that the use of dual-frequency ultrasonic irradiation and copper catalyst could significantly improve the reaction efficiency and yield; in particular, the yield could reach 75% when the ultrasound bath was combined with the probe. Although the yield under metal-free conditions was low, it still had regioselectivity, indicating that this method had certain applicability under different conditions. By combining the copper turnings and copper sulfate pentahydrate as catalysts with the ultrasound bath and probe, the yield could reach 75% within 60 min (Scheme 6) [39].

**Scheme 7:**
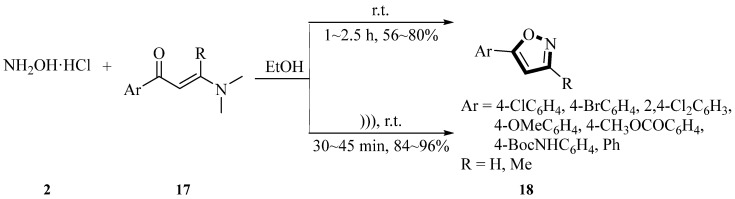
Synthesis of **18**.

In 2014, Huang et al. [40] synthesized a series of 5-arylisoxazole derivatives **18** via the reaction of 3-(dimethylamino)-1-arylprop-2-en-1-one **17** with hydroxylamine hydrochloride **2** under ultrasound irradiation using ethanol as the solvent. One of the most striking features of this reaction was that no catalyst was required. Under ultrasound irradiation, the yields of 5-arylisoxazole derivatives **18** ranged from 84% to 96%, whereas reactions without ultrasound irradiation provided lower yields (56~80%). The reaction time was significantly shortened to 30~45 min under sonication, compared to 1~2.5 h under conventional thermal conditions. The reaction temperature was controlled by adjusting the water level in the ultrasonic bath (via addition or removal of H_2_O), and the crude product was purified through recrystallization from ethanol. Under optimized conditions, various 5-arylisoxazole derivatives **18** were successfully synthesized, generating good yields regardless of whether the aromatic ring bore electron-withdrawing or electron-donating substituents. This method demonstrated advantages such as simple operation, mild reaction conditions, high yields, shorter reaction time, and an environmentally benign procedure (Scheme 7).

**Scheme 8:**
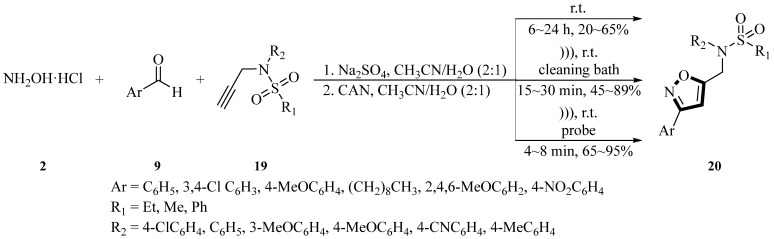
Synthesis of **20**.

In 2017, Alaoui and colleagues reported the synthesis of a novel sulfonamide–isoxazole compound **20** using a one-pot reaction of various aldehydes **6** and alkynes–sulfonamides **19** under ultrasonic radiation. The reaction was carried out at room temperature using a solvent mixture of H_2_O and acetonitrile (2:1 ratio), and ceric ammonium nitrate (CAN) was used as the key oxidizing agent. Hydroxylamine hydrochloride **2** was used as a precursor for imine formation, and sodium sulfate was used to facilitate the reaction. Under optimal reaction conditions, the sonication method could save 340 to 356 min compared with the conventional stirring method. For ultrasound assistance, two devices were used to increase the efficiency of the reaction: an ultrasonic scavenging bath (47 kHz) and an ultrasonic probe (20 kHz). The reaction could not proceed in the absence of an oxidizing agent, and when CAN was used as an oxidizing agent, the reaction in the ultrasonic scavenging bath took 20 min and yielded 88% of the product. The use of an ultrasonic probe reduced the reaction time to 4 min and increased the yield to 95%. Reducing the amount of CAN to 0.5 mmol resulted in incomplete conversion and lower yields. This one-pot synthesis strategy is carried out in an aqueous medium and uses inexpensive and environmentally friendly CAN as an oxidizing agent, in line with the principles of green chemistry. The process is simple, efficient and uses readily available starting materials, which are important for the further synthesis of 3,5-functionalized isoxazoles (Scheme 8) [41].

**Scheme 9:**
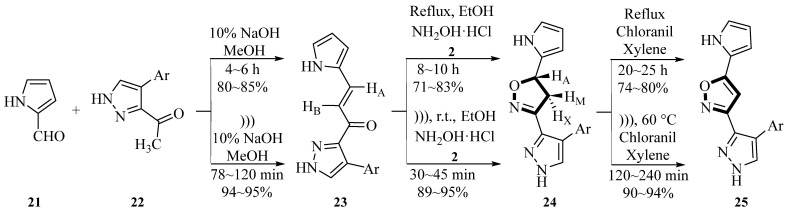
Synthesis of **25**.

In 2018, the group used an ultrasound-assisted reaction of pyrrole-2-carboxaldehyde **21**, 1-(4-aryl-1*H*-pyrazol-3-yl)ethan-1-one **22**, and hydroxylamine hydrochloride **2** to generate 3-(4-arylpyrazol-3-yl)-5-(pyrrol-2-yl)isoxazole **25** by using chloranil and xylene as mixed solvents. This was the first synthesis of a triheterocyclic system containing pyrrole, pyrazole, and isoxazole. They determined the antioxidant activity of the compounds **24** and **25** using DPPH, NO, and H_2_O_2_. Compound **25** exhibited significant activity in all three antioxidant activity tests, comparable to that of standard ascorbic acid, showing good antioxidant potential. The former of the two studies focused on green processes while the latter explored structural diversity and bioactivity; together, they advanced sonochemistry in organic synthesis (Scheme 9) [42].

**Scheme 10:**
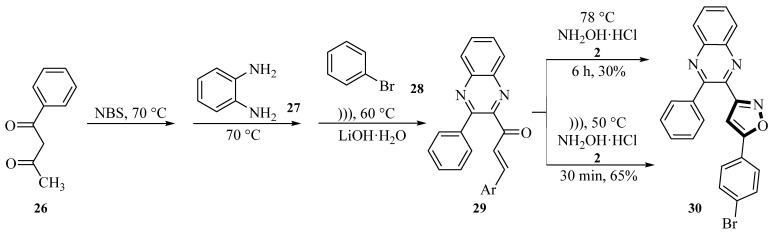
Synthesis of **30**.

In 2017, the Salman group demonstrated the formation of isoxazole derivative **30**, which was achieved by reacting chalcone-substituted quinoxaline derivative **29** with hydroxylamine hydrochloride **2** and sodium acetate under ultrasound irradiation. Hydroxylamine underwent a nucleophilic addition reaction with the α,β-unsaturated carbonyl groups of chalcone **26** to form an intermediate. The intermediate **29** was further cyclized to form an isoxazole ring. Ultrasound technology was used in two of these three reaction steps. The reaction process required heating to 50 °C in ethanol solvent for 85 min. The yield of the product obtained by this method was about 65%, which meant that the ultrasound-assisted method significantly improved the synthesis efficiency by about 35 percentage points. This strategy provided an efficient and green synthesis method, which has important reference value for the synthesis of nitrogen-containing heterocyclic compounds. The promotion of ultrasound-assisted synthesis technology is expected to be widely applied in the field of organic synthesis, especially in scenarios where reaction time needs to be shortened and product yield needs to be improved (Scheme 10) [43].

**Scheme 11:**
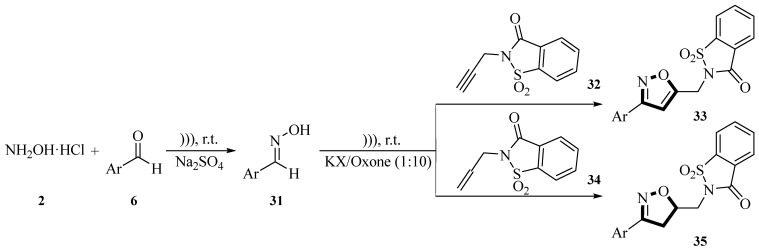
Synthesis of **33** and **35**.

In 2019, the Tachallait group described a method for the preparation of novel *N*-saccharin isoxazole derivative **33** and isoxazoline derivative **35** using KI/Oxone as a catalyst through a sonication-assisted cycloaddition reaction of hydroxylamine hydrochloride **2**, aldehydes **6**, and dipolarophiles promoted in water solvent. This is a one-pot three-step reaction utilizing in situ-generated nitrile oxides and dipolarophiles via a 1,3-dipolar cycloaddition reaction. The first step generates an oxime **31**, the second generates a nitrile oxide, and the third undergoes a 1,3-dipolar cycloaddition to generate the target product **33** and **35**. It is important to note that the first two steps do not require ultrasound, but the second step requires Oxone as the oxidizing agent. The third step in the reaction was carried out at room temperature for 30 min and the exact structure of the product depends on the aldehyde and olefin/alkyne substrate used. The yields ranged from 65% to 85% for isoxazoles **33** and 70% to 95% for isoxazoline **35**, indicating that the synthetic method is highly efficient. This reflects the advantages of this green method for the synthesis of *N*-sulfonamide isoxazole and isoxazoline derivatives. The method used H_2_O as the solvent, avoiding the use of volatile organic solvents and conforming to the principles of green chemistry (Scheme 11) [44].

**Scheme 12:**
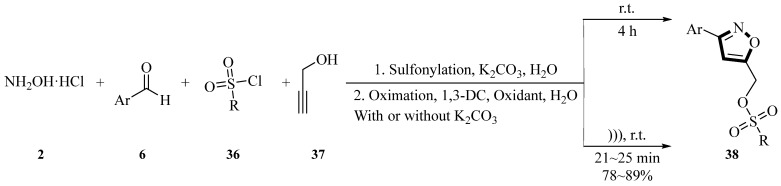
Synthesis of **38**.

In 2021, Talha and co-workers reported a one-pot four-component synthesis of 3,5-disubstituted isoxazole-sulfonates and -sulfonamides **38** using hydroxylamine hydrochloride **2**, aromatic aldehydes **6,** sulfonyl chloride **36,** and propargyl alcohol/amine **37** in water solvent under ultrasound irradiation. The reaction employed sodium dichloroisocyanurate (NaDCC) as a metal-free catalyst and oxidant, combined with ultrasonic activation (47 kHz) at room temperature, achieving completion within 20~28 min. This green methodology utilized H_2_O as the solvent, avoided toxic organic reagents, and demonstrated broad substrate compatibility, with a product yield of 72~89% depending on the substrate combination. The process proceeded via in situ sulfonylation, oximation, and 1,3-dipolar cycloaddition steps, generating four new bonds (S-O, C-N, C-O, and C-C). Compared with conventional magnetic stirring, ultrasound irradiation significantly enhanced reaction efficiency by improving mass transfer and reducing dimerization side reactions. This study not only provides an environmentally benign route for synthesizing isoxazole derivatives but also highlights the synergistic potential of ultrasound and heterogeneous catalysis in green chemistry and pharmaceutical development (Scheme 12) [45].

In recent years, ultrasound-assisted multicomponent reactions have emerged as powerful tools for sustainable heterocyclic synthesis. In 2022 and 2024, the Bougrin group published two articles describing the generation of isoxazole derivatives **42** in a one-pot, five-component reaction. In the 2022 article, they pioneered a one-pot five-component strategy using CaCl_2_/K_2_CO_3_ as a synergistic catalytic system to synthesize 3,5-disubstituted isoxazole secondary sulfonamides **42** from hydroxylamine hydrochloride **2**, aromatic aldehydes **6**, primary amines **39**, propargyl bromide **40,** and saccharin **41**. Their aqueous-phase protocol (optimization from MeOH/H_2_O 4:1 mixed solvent system to pure H_2_O system) employed ultrasonic cavitation (sonotrode: 20 kHz, 130 W; bath: 37 kHz, 280 W) at 25 °C with K_2_CO_3_ (3~4 mmol) and Oxone (2 mmol), achieving 75~96% yields within 13~17 min while emphasizing advantages of ultrasound-assisted reaction (Scheme 12) [46].

**Scheme 13:**
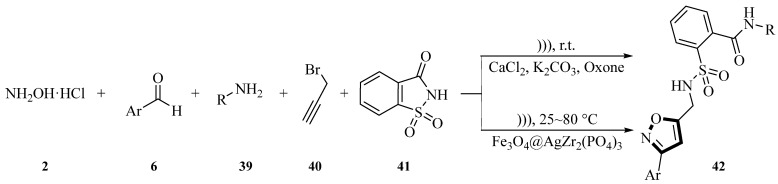
Synthesis of **42**.

Building on this, they developed an advanced Fe_3_O_4_@AgZr_2_(PO_4_)_3_ nanocatalyst for a one-pot four-step cascade synthesizing isoxazole-linked 1,2,3-triazoles **42** from propargyl alcohols, benzenesulfonyl chloride, aryl aldoximes, sodium azide, and terminal alkynes in 2024. Their ultrasound-assisted aqueous system (20 kHz, 60% power) demonstrated remarkable efficiency improvements, reducing reaction time from 24 h to 75 min (135 min total) and boosting yields from 79% to 94% under temperature gradient conditions (25~80 °C). This ultimately resulted in 86% to 94% yields of all synthesized compounds **42** in a relatively short reaction time (135 min). Both protocols highlight ultrasonic cavitation’s dual role in enhancing reaction kinetics and selectivity while employing H_2_O as a green solvent. The Mokhi system further advanced sustainability through magnetic catalyst recyclability (five cycles without significant activity loss), contrasting with Mahmoudi’s homogeneous base system (Scheme 13) [47]. These methodologies collectively exemplify progress in atom-economical heterocycle synthesis, addressing traditional limitations of toxic reagents, energy-intensive conditions, and poor regiocontrol through innovative catalyst design and mechanochemical activation.

### 2.2. Synthesis of Benzisoxazole-Based Derivatives

Benzisoxazole derivatives demonstrate significant therapeutic value due to their rigid bicyclic scaffold, enabling precise target engagement. Clinically, they form the core of antipsychotics like risperidone and iloperidone. Additional activities include potent antimicrobial (particularly antitubercular), anticonvulsant (via GABAergic modulation), antiglycation (for diabetic complications), and anticancer effects (kinase inhibition/apoptosis induction). Their structural versatility supports multitarget drug development. However, there are few organic synthesis programs based on ultrasound-assisted methods, which need to be further developed [48].

**Scheme 14:**
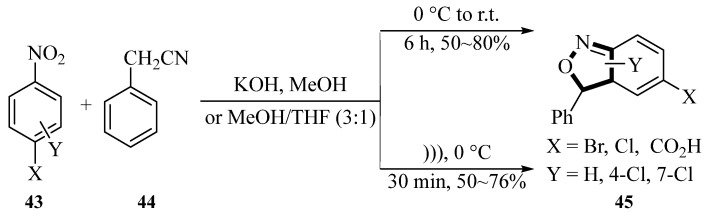
Synthesis of **45**.

In 2007, the Safaei-Ghomi group described a method of generating 2,1-benzisoxazole derivatives **45** through ultrasonic irradiation in the process of synthesizing 2-aminobenzophenone derivatives via nucleophilic substitution of nitrobenzene derivatives **43** with phenylacetonitrile **44**. The effect of ultrasound on organic reactions was attributed to cavitation, a physical process that creates, enlarges, and implodes gaseous and vaporous cavities in an irradiated liquid, resulting in very high local temperatures and pressure inside the bubbles (cavities), leading to turbulent flow in the liquid and enhanced mass transfer. They found that this physical process not only demonstrated the advantage of accelerating the reaction process with ultrasound but also improved the yield in shorter reaction times. The reaction for preparing 2,1-benzisoxazoles **45** required the use of MeOH/THF(3:1) as a solvent and maintenance of low temperature (0 °C) in an ice bath, which required only 30 min under ultrasound conditions (46 kHz, 200 W) (Scheme 14) [49].

### 2.3. Synthesis of Isoxazolone-Based Derivatives

Isoxazolone derivatives show broad bioactivity potent antifungal, antitubercular, and antileprotic agents; serve as peptide mimetics for conformationally restricted drug design; and act as key enzyme inhibitors (e.g., aldose reductase). Their structural versatility enables development of novel antimicrobials and therapeutic tools, particularly against neglected tropical diseases [50].

In synthesis, 5-isoxazolone derivatives act as key intermediates for constructing bioactive molecules. Materials applications leverage their rigid structure for photochromic devices, energy materials, and liquid crystals. Agriculturally, isoxazoline-based insecticides target GABA receptors. Their structural adaptability drives innovation across these fields [33].

**Scheme 15:**
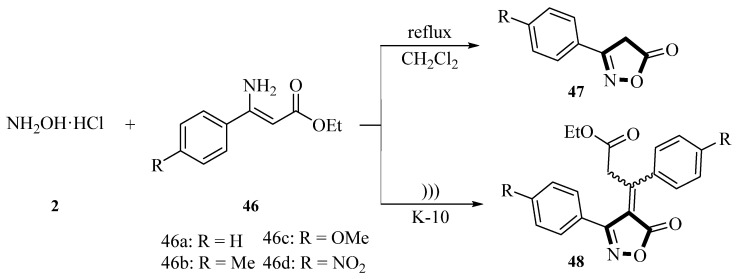
Synthesis of **47** and **48**.

In 1999, the Valduga group also reported a method for ultrasound-assisted generation of isoxazolone derivatives. For the β-enamino esters **46**, divergent pathways were observed: Under conventional reflux in dichloromethane for 20 h, compounds **46** reacted to form the expected 3-(4-phenyl-substituted)-4,5-dihydro-5-isoxazolones **47** with yields of 58% to 75%, while **46d** remained unreacted. However, employing K-10/ultrasound under identical solvent and temperature conditions for 6 h resulted in the formation of novel ethyl 3-[(4-phenyl-substituted)-5-oxo-4,5-dihydro-4-isoxazolyliden]-3-(4-phenyl-substituted)propanoates **48** as mixtures of E- and Z-isomers, with yields ranging from 56% to 80%. This ultrasound-assisted method activated the unreactive substrate **46d**, shortened the reaction time significantly (from 20 h to 6 h), and provided access to rare 5-oxoisoxazolylidene derivatives (Scheme 15) [34].

**Scheme 16:**
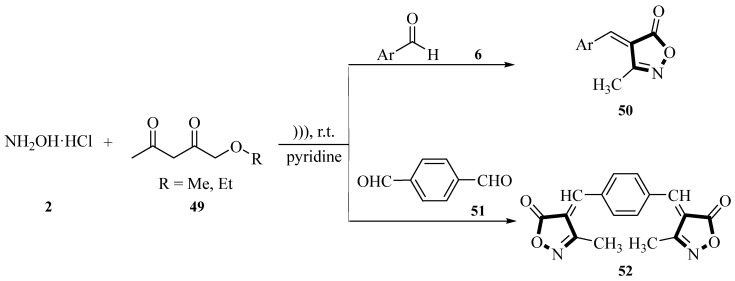
Synthesis of **50** and **52**.

In 2009, the Cheng group reported a method for synthesizing 3-methyl-4-arylmethylene-isoxazol-5(4*H*)-one derivatives **50** through a three-component one-pot condensation reaction of hydroxylamine hydrochloride **2**, aromatic aldehydes **6** (such as terephthalaldehyde), and methyl (or ethyl) acetoacetate **49** in aqueous media under ultrasonic irradiation. The reaction employed pyridine as an acid-binding agent to neutralize the hydrochloric acid released from hydroxylamine hydrochloride **2**, maintaining a neutral or weakly basic environment, thereby reducing side reactions and improving reaction selectivity. Experimental results showed that the reaction proceeded smoothly at room temperature with moderate to good yields (51~96%) regardless of whether the aryl ring was attached to electron-donor groups such as *p*-dimethylamino and *p*-methoxy, or to electron-withdrawing groups such as *p*-nitro and *p*-fluoro. The ultrasonic irradiation time ranged from 30 to 90 min. The reaction also proceeded well for ortho-substituted aldehydes, α,β-unsaturated aldehydes, and heteroaromatic aldehydes, with yields ranging from 47% to 71%. Additionally, terephthalaldehyde **51** underwent two condensation reactions with methyl acetoacetate **49** and hydroxylamine hydrochloride **2** to form the bis-isoxazolone derivative **52**, with a yield of 63% after 90 min. It was experimentally demonstrated that the method is environmentally friendly (aqueous medium), which provides an efficient and green synthesis route for isoxazolone derivatives (Scheme 16) [51].

**Scheme 17:**
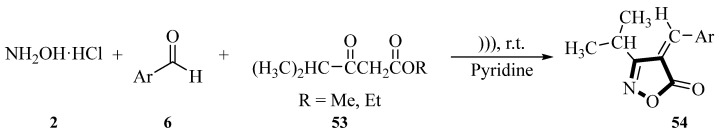
Synthesis of **54**.

In 2012, the Ablajan group described a three-component, one-pot reaction of methyl hydroxylamine hydrochloride **2**, aromatic aldehydes **6**, and 4-methyl-3-oxovalerate **53**, to synthesize 4-arylmethylidene-3-isopropylisoxazol-5-ones **54** under ultrasonic irradiation, catalyzed by pyridine in an aqueous medium at room temperature. The study highlighted the advantages of multicomponent reactions (MCRs) in organic synthesis, including straightforward procedures, experimental simplicity, and high product yields (63~96%). The reaction sequence involved an initial 5 min ultrasonic activation of the reactants (methyl 4-methyl-3-oxovalerate **53**, hydroxylamine hydrochloride **2** and pyridine) in H_2_O, followed by the addition of aromatic aldehyde **6** and continued ultrasonic irradiation for 40 min. Notably, the aqueous solvent system is aligned with green chemistry principles as it minimizes environmental impact and operational costs. This experiment confirmed the formation of targeted isoxazol-5-ones **54** using structural characterization while maintaining the advantages of the sonication reaction, in which electron-withdrawing substituents on aromatic aldehydes enhance the reactivity (Scheme 17) [52].

**Scheme 18:**
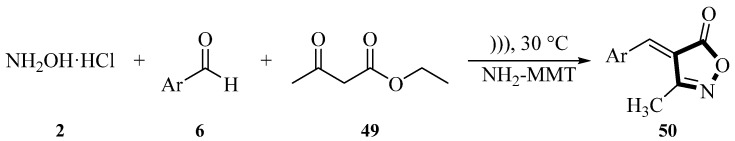
Synthesis of **50**.

In 2016, the Safari group described an ultrasound-assisted reaction for synthesizing 3-methyl-4-arylmethylene isoxazole-5(4*H*)-ones **50** using hydroxylamine hydrochloride **2**, benzaldehyde derivatives **6**, and ethyl acetoacetate **49** under ultrasound irradiation conditions with NH_2_-MMT as the catalyst. MMT-K10 has received considerable attention as a catalyst and catalyst carrier in chemical synthesis. The group modified MMT by introducing 3-aminopropyltriethoxysilane (APTES), prepared NH_2_-MMT, and used it as a nanocatalyst for chemical reactions. The reaction was conducted at 30 °C using H_2_O as the solvent and NH_2_-MMT as the catalyst, under irradiation with an ultrasonic power of 100 W. The reaction time varied between 10 and 55 min and the yield ranged from 80% to 97% depending on the substrate. The ultrasound-assisted synthesis method using NH_2_-MMT as a catalyst had a shorter reaction time, easier post-treatment, higher yield, and a simpler experimental process. NH_2_-MMT exhibited good recovery and reuse in the 6-cycle model reaction, with only a slight decrease in yield (Scheme 18) [53].

**Scheme 19:**
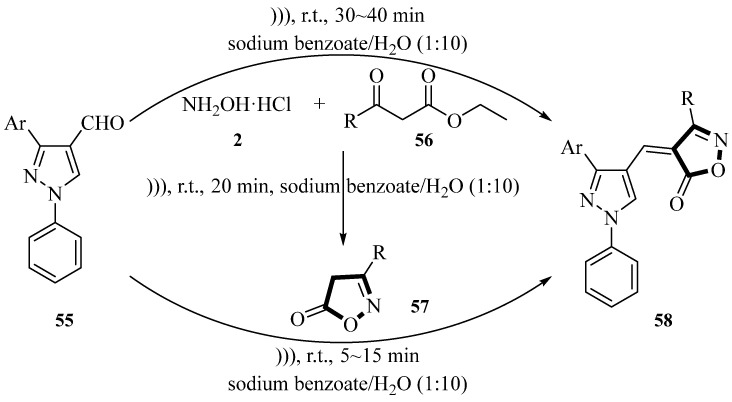
Synthesis of **58**.

In 2017, the Konkala group published an article on the reaction of hydroxylamine hydrochloride **2**, pyrazole-4-carbaldehyde **55**, and β-ketoesters **56** to generate 4-pyrazolylmethylene-isoxazol-5(4*H*)-ones derivatives **58** catalyzed by sodium benzoate using sonochemical methods. During the process, they first synthesized the starting material 1,3-diphenyl-1*H*-pyrazole-4-carbaldehyde **57** via the Vilsmeier–Haack reaction, and then used this compound as the substrate for the one-pot three-component cyclocondensation reaction with the other two reactants. They screened various catalysts and solvents for the reaction. Experimental results indicated that only sodium benzoate, piperidine, and sodium acetate efficiently catalyzed the reaction, with sodium benzoate providing the highest yield (88%) in H_2_O under ultrasonic irradiation. The reaction was conducted at room temperature, and the one-pot and step-by-step methods had similar times and yields. The one-pot method offers advantages such as operational simplicity, shorter reaction time, and scalability for industrial applications. The step-wise approach, though involving intermediate isolation, is suitable for mechanistic studies and reaction optimization. This protocol aligns with green chemistry principles by avoiding hazardous organic solvents, utilizing H_2_O as a reaction medium, and enabling catalyst recycling. The method also features high efficiency, minimal by-products, and compatibility with diverse substrates, making it a viable strategy for Knoevenagel condensation reactions (Scheme 19) [54].

**Scheme 20:**
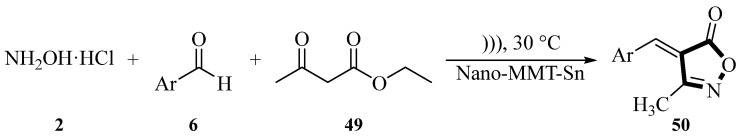
Synthesis of **50**.

In 2018, an ultrasound-assisted method for synthesizing 3-methyl-4-(hetero)arylmethylene isoxazole-5(4*H*)-ones **50** was reported by Ahmadzadeh et al. [55]. The target compounds **50** were synthesized via a one-pot multicomponent cyclocondensation of hydroxylamine hydrochloride **2**, aromatic aldehydes **6**, and ethyl acetoacetate **49** catalyzed by Sn^II^-Mont K10. The catalyst exhibited recoverability through simple filtration and maintained consistent catalytic activity over six reuse cycles. Reaction optimization revealed the optimal conditions to be 0.01 g of catalyst, 90 W ultrasonic power, 30 °C temperature, and 20 min reaction time in an aqueous medium, achieving a maximum yield of 96%. Substrates with electron-donating or electron-withdrawing groups demonstrated yields ranging from 87% to 96%. This study pioneered the synergistic combination of Sn^II^-Mont K10 with ultrasound irradiation, leveraging cavitation effects to enhance reaction efficiency under mild, solvent-free conditions. The methodology emphasized green chemistry principles by eliminating organic solvents, reducing energy consumption, and enabling catalyst reuse, offering a novel approach for sustainable heterocyclic compound synthesis (Scheme 20).

**Scheme 21:**
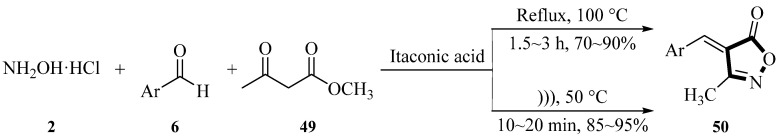
Synthesis of **50**.

In 2019, the Kasar group reported a method of synthesizing 4*H*-isoxazol-5-ones derivatives **50** via a multicomponent reaction of hydroxylamine hydrochloride **2**, aromatic aldehydes **6**, and ethyl acetoacetate **49** catalyzed by itaconic acid under ultrasonic irradiation. Optimization studies revealed that under conventional heating (100 °C, 3 h), the reaction achieved a 90% yield. In contrast, ultrasound-assisted conditions (50 °C, 15 min) significantly enhanced efficiency, delivering a 95% yield. Notably, the aqueous solution of itaconic acid could be reused for ten catalytic cycles without loss of activity. The protocol employs H_2_O as a green solvent and ultrasonication as a non-conventional energy source, aligning with green chemistry principles by eliminating organic solvents, hazardous acids, and metal catalysts. This method demonstrates high efficiency, broad substrate compatibility, and excellent catalyst reusability, providing a sustainable approach for synthesizing biologically active isoxazole derivatives (Scheme 21) [56].

**Scheme 22:**
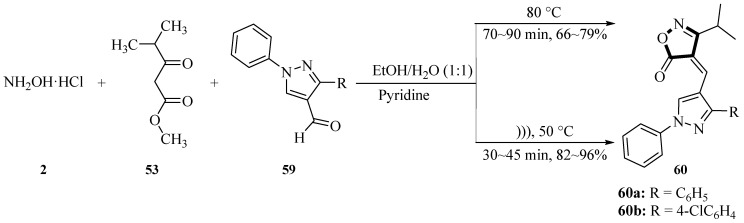
Synthesis of **60**.

In 2019, Bhatt and co-workers synthesized 4-(substituted-1*H*-pyrazol-4-yl) methylene)-3-isopropylisoxazol-5(4*H*)-ones **60** via a one-pot, three-component reaction using hydroxylamine hydrochloride **2**, methyl 4-methyl-3-oxovalerate **53** and substituted pyrazole aldehyde **59** under ultrasonic irradiation conditions. The ultrasound-assisted method (300 W, 20~60 kHz) was conducted at 50 °C for 30~45 min, with a product yield of 82~96% with pyridine as the base. The synthesized compounds were evaluated for in vitro anticancer activity, demonstrating significant growth inhibitory effects against various tumor cell lines, particularly leukemia cell lines. Compared with the conventional heating method (70~90 min, 66~79% yield), ultrasonic irradiation provided shorter reaction times (25~60 min), higher yields (3~30%), and reduced impurity formation (Scheme 22) [57]. The synthesized compounds **60** were evaluated for their in vitro anticancer activity using a single high dose (10 μM) against a panel of 60 human tumor cell lines derived from nine cancer types. The results showed that most of the compounds exhibited significant growth inhibitory activity against human tumor cells, particularly the leukemia cell lines, which showed remarkable sensitivity to compounds **60a** and **60b**. Compound **60a** demonstrated the most potent cytotoxic activity, especially against leukemia (HL-60(TB); GI_50_ = −45.19), melanoma (LOX IMVI; GI_50_ = 17.99), and colon cancer (HCT-116; GI_50_ = 28.89) cell lines.

**Scheme 23:**
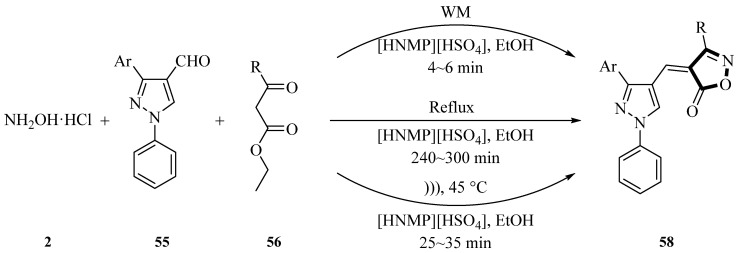
Synthesis of **58**.

In 2020, the Shirole group investigated the reaction of 1,3-diaryl-1*H*-pyrazole-4-carboxyaldehyde **56**, β-keto ester **55**, and hydroxylamine hydrochloride **2** to form 3-methyl-4-((3-aryl-1-phenyl-1*H*-pyrazol-4-yl)methylene)isoxazol-5(4*H*)-one derivatives **58** via a one-pot multicomponent reaction catalyzed by the ionic liquid [HNMP][HSO_4_]. The reaction was conducted under both ultrasound irradiation (45 °C, 30 min) and microwave irradiation (210 W, 5 min), achieving yields of 80~82% for the target products. The authors first optimized the model reaction by determining the optimal catalyst dosage (100 mg) and solvent (ethanol), with the conventional reflux method having a product yield of 70% under optimized conditions. This study emphasizes the typical advantages of ultrasound-assisted methods and highlights their importance in the field of sustainable chemical synthesis and drug development (Scheme 23) [58].

**Scheme 24:**
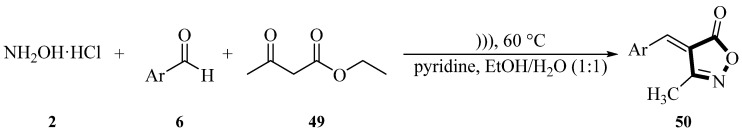
Synthesis of **50**.

In 2021, the Ren group investigated a method for the synthesis of 3-methyl-4-aromatic methylene isoxazole-5(4*H*)-ketone **50** using ultrasonic radiation, with hydroxylamine hydrochloride **2**, aromatic aldehydes **6**, and methyl acetoacetate **49** as substrates, and pyridine as a catalyst to accelerate the reaction. The one-pot preparation simplified the post-reaction treatment steps. Results showed that a 96% yield was obtained with *p*-dimethylaminobenzaldehyde as the aromatic aldehyde **6**, while a 64% yield was achieved with benzaldehyde **6**. By comparing the effects of different aromatic aldehydes on product yields, it was observed that aromatic aldehydes **6** with electron-donating groups provided higher yields, whereas those with electron-withdrawing groups resulted in lower yields. During their experiments, they found that the ethanol–water system reacted faster than pure water. The sonication reaction required approximately 60 min at 60 °C, with a product yield of 43~96%. The incorporation of ultrasound technology significantly simplified operations and improved yields while maintaining environmental friendliness; however, this method imposed limitations on solvent choices and generated minor by-products. The application of ultrasonic radiation in organic synthesis could be further improved through the optimization of reaction conditions, exploration of more efficient catalysts, and implementation of real-time monitoring tools (Scheme 24) [59].

**Scheme 25:**
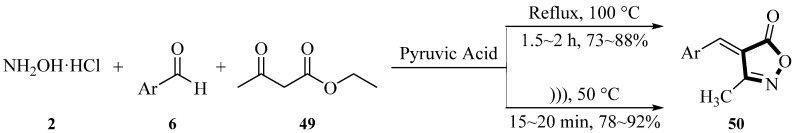
Synthesis of **50**.

In 2022, the Deshmukh group reported a one-pot three-component synthesis of 3-methyl-4-arylmethylene isoxazol-5(4*H*)-ones **50** using hydroxylamine hydrochloride **2**, benzaldehyde derivatives **6**, and ethyl acetoacetate **49** in aqueous medium catalyzed by pyruvic acid (5 mol%). The reaction mechanism involves pyruvic acid-mediated activation of ethyl acetoacetate **49**, followed by oxime formation, keto-enol tautomerization, nucleophilic attack on the aldehyde **6**, intramolecular cyclization, and deprotonation to yield the isoxazole **50** scaffold. They found that ultrasonic irradiation reduced the temperature by 50 °C compared to conventional heating methods; the formation rate increased by only 4%, but the time was reduced to one-tenth of that of the conventional method. The protocol demonstrated broad substrate compatibility, accommodating mono- and di-substituted aromatic aldehydes **6**, with a product yield of 73~92%. Key innovations include the use of H_2_O as a green solvent, biodegradable pyruvic as an acid catalyst, and ultrasound technology to enhance efficiency. This method avoids the use of toxic reagents and highlights its potential in the green synthesis of bioactive isoxazole derivatives (Scheme 25) [60].

**Scheme 26:**
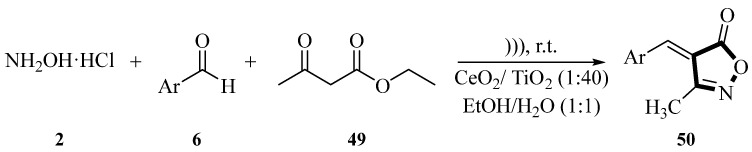
Synthesis of **50**.

In 2022, Maddila and his colleagues published a simple and efficacious preparation of isoxazole-5(4H)-one analogues **50** via multicomponent, single-step condensation of hydroxylamine hydrochloride **2,** substituted aldehyde **6**, and ethyl acetoacetate **49** in the presence of a recyclable catalyst under ultrasound-assisted reaction at room temperature. The reaction showed various benefits like easy work-up procedure, short reaction times (not more than 10 min), use of greener solvents, lack of toxic reagents, excellent product yields (not less than 94%), and recyclability. The main advantage of this method was that the solid catalyst was utilized for over eight cycles without any significant loss of activity (Scheme 26) [61].

**Scheme 27:**
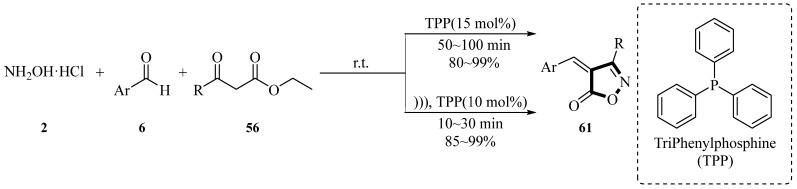
Synthesis of **61**.

In 2023, Daroughehzadeh and Kiyani reported a three-component organocatalytic approach for the synthesis of 4-arylideneisoxazol-5(4*H*)-ones **61** using triphenylphosphine (TPP) as a catalyst. The model reaction involved hydroxylamine hydrochloride **2**, 4-hydroxy-3-methoxybenzaldehyde **6**, and ethyl acetoacetate **56** under aqueous conditions. Optimization studies revealed that 15 mol% TPP at 25 °C in H_2_O afforded the target product a 98% yield within 50 min. Sonochemical synthesis further enhanced the reaction efficiency, achieving a 99% yield with only 10 mol% TPP in 10 min under ultrasonic irradiation. Comparative analysis demonstrated the superiority of ultrasonication in reducing reaction time and catalyst loading. Notably, the study emphasized the green aspects of the protocol, including H_2_O as a solvent and high atom economy, but did not report catalyst recyclability data. This work highlights the efficacy of TPP in multicomponent heterocyclization and the benefits of sonochemical activation (Scheme 27) [62].

**Scheme 28:**
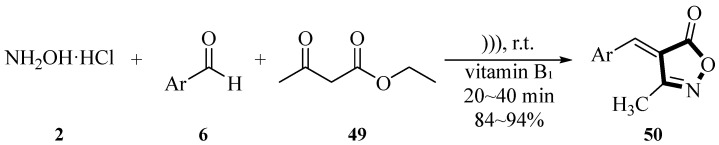
Synthesis of **50**.

In 2023, the Zhang group developed an efficient method for the synthesis of 3-methyl-4-(arylmethylene)isoxazol-5(4*H*)-ones **50** via a vitamin B_1_-catalyzed cyclocondensation reaction under ultrasound radiation. The model reaction system employed hydroxylamine hydrochloride **2**, 2-methoxybenzaldehyde **6**, and ethyl acetoacetate **49** in H_2_O as the solvent. Nucleophilic attack of hydroxylamine hydrochloride **2** on the ethyl acetoacetate **49** carbonyl group first leads to the formation of an oxime intermediate via dehydration. Next, vitamin B_1_-mediated activation of the aldehyde group occurs in a Knöwenagel condensation reaction with the tautomerized intermediate. Finally, intramolecular cyclization and elimination of ethanol produces the target compound. The reaction achieved a 94% yield under optimized conditions (0.1 equiv vitamin B_1_, 20 °C, 30 min). Ultrasound-assisted synthesis significantly improved yields (84~94%) and reduced reaction time by 85% compared to conventional stirring. Notably, vitamin B_1_ exhibited excellent reusability, maintaining yields above 88% after five cycles. This method was metal-free, acid/base-free, and environmentally friendly, leveraging H_2_O as a green solvent (Scheme 28) [63].

**Scheme 29:**
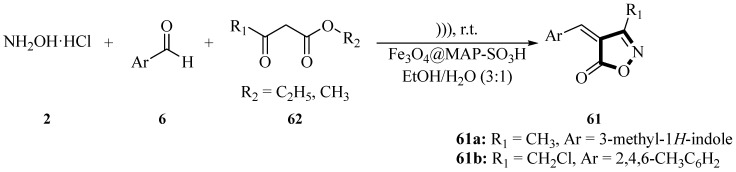
Synthesis of **61**.

In 2023, the Nongrum group described a three-component condensation reaction comprising hydroxylamine hydrochloride **2**, aromatic aldehydes **6**, and β-ketoesters **62** using Fe_3_O_4_@MAP-SO_3_H as a catalyst to synthesize 3-methyl-4-(phenyl)methylene-isoxazole-5(4*H*)-one derivatives **61**. The reaction was mediated by ultrasound irradiation in an ethanol–water (1:3) medium under a one-pot protocol. Optimization studies indicated that 20 mg of catalyst in an ethanol–water (1:3) medium under ultrasound irradiation provided the highest yield (92%), significantly accelerated the reaction rate and reaction time to 20 min. The synthesized compounds **61** were evaluated for antifungal and antimycobacterial activity. The 3-chloromethyl-4-(phenyl)methylene-isoxazole-5(4*H*)-one series (notably compound **61a**) exhibited over 50% growth inhibition against Fusarium oxysporum at 100 µg/mL. Compounds **61a** and **61b** displayed antimycobacterial activity against Mycobacterium tuberculosis H37Rv strain, with MIC values (1.56 µg/mL) equipotent to the standard drug ethambutol. Additionally, these compounds demonstrated efficacy against both active and dormant forms of *M. tuberculosis* in a nutrient starvation model. This green synthesis method features easy catalyst recovery via an external magnet and the use of eco-friendly solvents. The biological activities of the compounds highlight their potential as candidates for antifungal and antimycobacterial drug development (Scheme 29) [64].

**Scheme 30:**
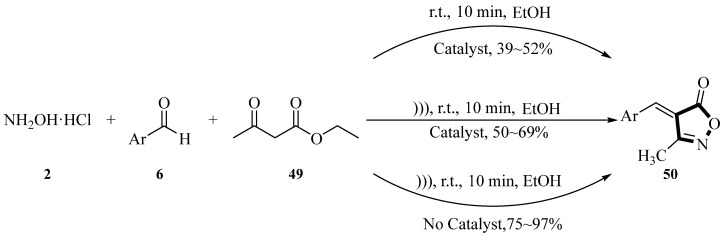
Synthesis of **50**.

In 2023, the Bankuru group developed a catalyst-free green protocol for synthesizing methyleneisoxazole-5(4*H*)-ones 50 using ultrasound irradiation. The researchers employed a one-pot, three-component reaction of ethyl acetoacetate **49**, aromatic aldehydes **6**, and hydroxylamine hydrochloride **2** in ethanol at ambient temperature. Ultrasonic irradiation (40 kHz, 100 W) was applied for ≤10 min, eliminating the need for catalysts. Optimization studies confirmed ethanol as the optimal solvent and 100 W as the ideal ultrasonic power, achieving excellent yields (92~98%) within 10 min across diverse substituted aldehydes **6**. Key advantages include operational simplicity, waste reduction, energy efficiency, and avoidance of toxic reagents or laborious purification. This method represents a significant advancement in sustainable heterocyclic synthesis by combining catalyst-free conditions with ultrasonic efficiency (Scheme 30) [65].

**Scheme 31:**
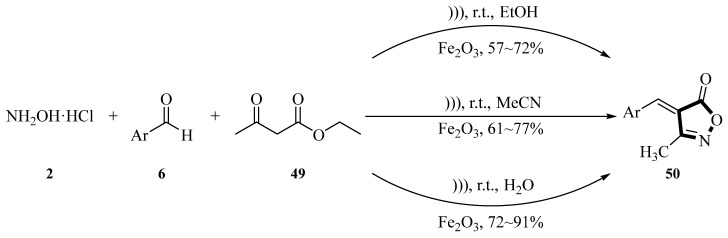
Synthesis of **50**.

In 2025, the Ghevade group developed an ultrasound-promoted ferrite-catalyzed protocol for synthesizing 3,4-disubstituted isoxazole-5(4*H*)-ones **50** in aqueous media. The methodology employed a one-pot multicomponent reaction of aromatic aldehydes **6**, ethyl acetoacetate **49**, and hydroxylamine hydrochloride **2** catalyzed by solgel synthesized ferrite nanoparticles (Fe_2_O_3_ NPs). Under optimized conditions (10 mol% catalyst, H_2_O solvent, 90 W ultrasonic irradiation, room temperature), the reaction achieved excellent yields (84~91%) within 20~35 min. This synthetic method eliminates the need for chromatographic purification, employs green solvents and highly efficient reusable catalysts, aligning with the principles of green chemistry. Synthesized compound **50** demonstrated significant antimicrobial activity against Staphylococcus aureus and Pseudomonas aeruginosa via well-diffusion assay. This work establishes Fe_2_O_3_ NPs as a sustainable nanocatalyst for ultrasound-enhanced synthesis of bioactive isoxazolones (Scheme 31) [66].

### 2.4. Synthesis of Isoxazoline-Based Derivatives

Isoxazoline derivatives exhibit multifunctional utility, as potent antitumor, anti-inflammatory, and neuroprotective/antidepressant effective GABA-targeting insecticides; versatile synthetic building blocks enabling stereoselective drug design; and antimicrobial/antiparasitics. Their structural adaptability supports broad therapeutic and agrochemical applications. Therefore, there are more cases of ultrasonic methods applied in the synthesis of isoxazoline [33].

**Scheme 32:**
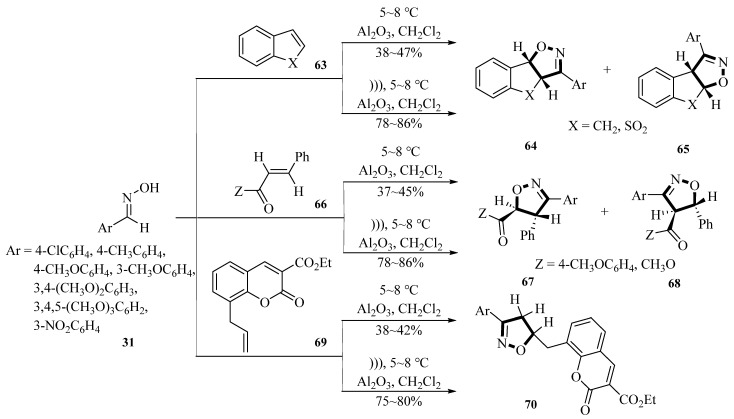
Synthesis of **64**, **65**, **67**, **68**, and **70**.

In 1999, the Syassi group published an article about synthesizing 4,5-dihydroisoxazoles derivatives **64**, **65**, **67**, **68,** and **70** using 1-sodium-3,5-dichloro-s-triazine-2,4,6-trione (SDCTT), arylaldoximes, and dipolarophiles under ultrasound irradiation conditions. The reaction utilized aluminum oxide as a catalyst and dichloromethane as a solvent. This method was activated by ultrasound in a solid–liquid two-phase system. They developed a new halogenating and oxidizing agent called SDCTT for the synthesis of the target compound. Arylaldoximes were used as substrates. The water bath temperature was maintained at 5~8 °C, and ultrasonic irradiation (47 kHz, 70 W) was applied for 15 min. Ultrasonic radiation had a significant impact on yield improvement. Specifically, ultrasound radiation increased the reaction yield from 37~47% under magnetic stirring to 75~86%. This indicated that ultrasonic radiation highly increased the synthesis yield of 4,5-dihydroisoxazoles, with an average yield increase of approximately 41.5%. This demonstrates the advantages of the ultrasound method (Scheme 32) [67].

**Scheme 33:**
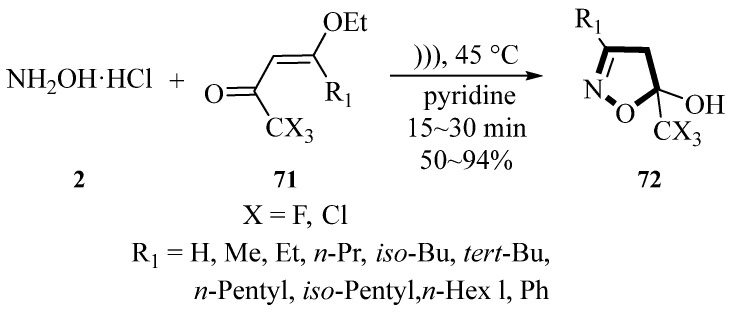
Synthesis of **72**.

In 2006, the Martins group described a reaction in which 1,1,1-trihalo-4-alkoxy-3-alken-2-ones reacted **71** with hydroxylamine hydrochloride **2** and pyridine to generate 5-hydroxy-5-trihalomethyl-4,5-dihydroisoxazoles **72** under ultrasound irradiation in a water bath at 45 °C. Under ultrasound irradiation conditions (20 W), the yields of 5-hydroxy-5-trihalomethy1-4,5-dihydroisoxazoles **72** ranged between 50% and 94%, and the reaction time was 30 min. The use of ultrasound irradiation utilized water as a solvent, thereby reducing environmental impact and cost. The product **72** had high purity and did not require further purification (Scheme 33) [68].

**Scheme 34:**
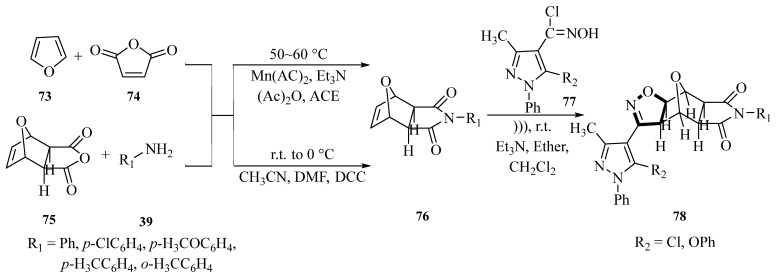
Synthesis of **78**.

In 2006, the Jia group described a novel procedure for preparing a series of unreported 3-(5-chloro/phenoxy-3-methyl-1-phenyl-4-pyrazolyl)isoxazole-*N*-substituted phenyl-dehydronorcantharidimide derivatives **78** via ultrasonic-assisted synthesis. The synthesis involved two key steps: first, the reaction of dehydronorcantharidin **75** with substituted aromatic amines **39** to form *N*-substituted phenyl-dehydronorcantharidimide intermediates **76**, and then the 1,3-dipolar cycloaddition of these intermediates with 5-chloro/phenoxy-4-(*α*-chloro-*α*-oximidomethyl)-3-methyl-1-phenylpyrazole **77** under ultrasonic conditions. Notably, the ultrasonic-assisted method was applied only in the second step (target product formation), significantly improving reaction efficiency: the average reaction time was reduced by 4~5 h, and yields increased from 30~50% (conventional method) to 65.9~81.3% (ultrasonic method). The reaction utilized anhydrous ether and dichloromethane as solvents under ultrasound irradiation at 40 °C for 10~15 min. This study provided an efficient approach to synthesizing novel pyrazole-containing isoxazole derivatives **78**, which might exhibit potential antitumor and other biological activities, offering valuable leads for future drug development (Scheme 34) [69].

**Scheme 35:**
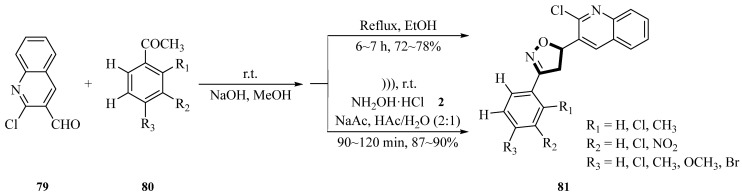
Synthesis of **81**.

In 2011, Tiwari et al. [70] presented an improved synthesis method for the preparation of 5-(2-chloroquinolin-3-yl)-3-phenyl-4,5-dihydroisoxazolines **81** using aqueous acetic acid under ultrasound irradiation, with chalcones and hydroxylamine hydrochloride **2** as substrates and sodium acetate as a base. The reaction was conducted at room temperature for 90~120 min under ultrasound irradiation (40 kHz, 250 W) in an ultrasonic cleaner. The optimized molar ratio of chalcone to hydroxylamine hydrochloride **2** was 1:3, yielding target compounds **81** with 80~90% efficiency. Ultrasonic irradiation shortened the process to 240~330 min, increased the yield compared with the conventional thermal method (72~78%) and its potential for practical applications (Scheme 35).

**Scheme 36:**
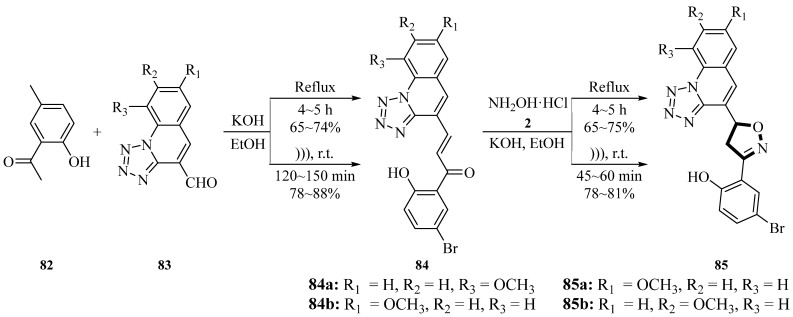
Synthesis of **85**.

In 2015, the Nikam group described a study in which a series of new isoxazoline derivatives **85** were synthesized from the corresponding chalcone **84** under ultrasonic radiation. The reaction was carried out in an ultrasonic bath at a frequency of 40 MHz and a power of 100 W. The reaction time was shortened from 2 h to a few min. The reaction yield for the generation of chalcone **84** was 78%, and the yields of isoxazoline derivatives **85** were 78~81%. Some of the synthesized compounds **84** and **85** showed significant biological antimicrobial and antioxidant activities, with compounds **84a** and **85a** having excellent antimicrobial activity, while compounds **84a**, **84b**, **85a**, and **85b** having excellent antioxidant activity, with IC_50_ values (21.0~26.5 μg/mL) surpassing those of ascorbic acid and BHT. Studies suggested interactions with fungal CYP51, and Absorption, Distribution, Metabolism, Excretion, and Toxicity (ADMET) predictions indicated favorable drug-like properties. This study provides a series of structurally diverse compounds with dual antimicrobial and antioxidant potential, offering promising leads for further development (Scheme 36) [71].

**Scheme 37:**
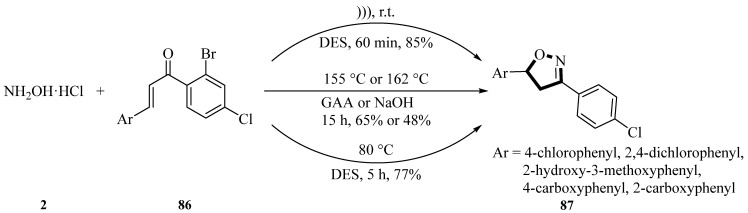
Synthesis of **87**.

In 2016, Bakht et al. [72] and co-workers developed an efficient method for the synthesis of isoxazoline derivatives **87** based on a DES prepared from benzalkonium chloride (BZK) and urea (1:2 molar ratio) in combination with ultrasonic irradiation (20 kHz, 130 W, 28~32 °C). The reaction was realized via cyclization of chalcone **86** with hydroxylamine hydrochloride **2**. In this study, benzalkonium chloride-based DES was used for the first time in organic synthesis with ultrasonication in a green chemistry-oriented approach. DES was produced by mechanically mixing benzalkonium chloride with urea for 30 min at room temperature. The reaction time was drastically shortened from 15 h to 1 h, the product **87** yield was increased from 48~65% to 78~85%, and the energy consumption was reduced by 86~88% compared with conventional solvents (glacial acetic acid/sodium hydroxide) as the reaction medium. Ultrasonication can accelerate molecular collision and mass transfer through the cavitation effect by generating localized high temperature (2000~5000 K) and high pressure (about 18,000 atm), which significantly improve reaction efficiency. In addition, DES showed good recyclability, with only a small decrease in yield after four cycles of reuse (Scheme 37).

**Scheme 38:**
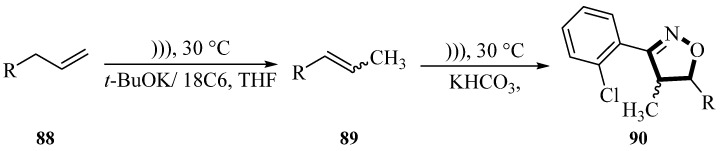
Synthesis of **90**.

In 2017, the Krompiec group reported highly active, regioselective, and reusable crown ether/base catalytic systems (e.g., 15-Crown-5/NaOH, 18-Crown-6/KOH, dibenzo-18-Crown-6/*t*-BuOK, and 18-Crown-6/*t*-BuOK) for double bond migration in allylic compounds **88** and **89**. These systems are capable of efficiently isomerizing a wide range of allyl substrates under mild conditions (typically 30 °C, 0.25~24 h). Remarkably, ultrasound assistance significantly accelerated reaction rates without compromising selectivity. The reactions proceeded in various solvents (THF, Et_2_O, DME, toluene, etc.) or under solvent-free conditions, with full regioselectivity observed for C,O- and O,S-diallyl compounds, where only the less substituted allyl group underwent migration. Additionally, a one-pot synthesis of isoxazolines **90** was demonstrated by combining isomerization, nitrile oxide generation, and 1,3-dipolar cycloaddition (Scheme 38) [73].

**Scheme 39:**
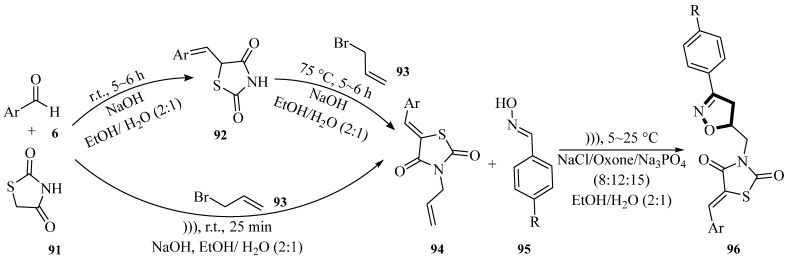
Synthesis of **96**.

In 2020, Thari and co-workers reported a method for the synthesis of isoxazoline derivatives **96** from aryl aldehydes **6**, thiazolidine-2,4-diones **91**, allyl bromide **93**, and sodium hydroxide via a one-pot, two-step reaction under ultrasonic irradiation, utilizing NaCl/Oxone/Na_3_PO_4_ in an aqueous medium as a chlorine source, oxidant, and catalyst. In the first step, *N*-allyl-5-arylidenethiazolidine-2,4-diones **94** were synthesized from thiazolidine-2,4-dione **91**, aryl aldehydes **6**, and allyl bromide **93** through Knoevenagel condensation followed by *N*-allylation using aqueous NaOH under ultrasonic conditions. This process avoids the separation of intermediates **92** required by conventional methods, and yields can be increased by 7% to 52% under ultrasonic conditions. The second step involved a regioselective 1,3-dipolar cycloaddition reaction using the NaCl/Oxone/Na_3_PO_4_ system under ultrasonic activation (130 W, 20 kHz) at 5~25 °C for 30 min in ethanol/water (2:1), yielding 69~90% of the target isoxazoline derivatives **96**. The catalyst system efficiently promoted cycloaddition while suppressing byproduct formation. Compared with traditional multi-step approaches, this ultrasound-assisted method simplifies the process, enhances reaction efficiency (24-fold acceleration in cycloaddition), and reduces environmental impact by employing aqueous media and recyclable reagents. The study presents an ecofriendly strategy for synthesizing *N*-thiazolidine-2,4-dione-isoxazoline hybrids with potential pharmacological applications, emphasizing improved selectivity and scalability (Scheme 39) [74].

**Scheme 40:**
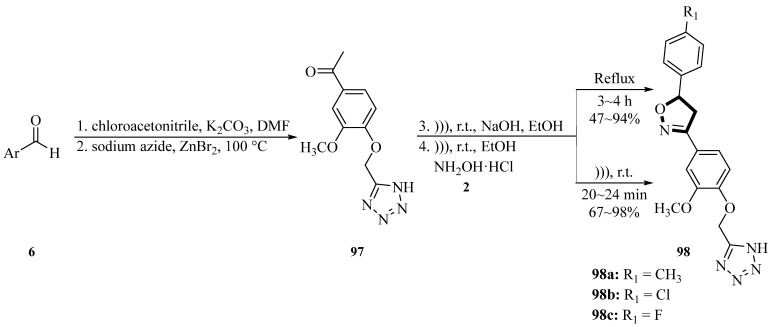
Synthesis of **98**.

In 2020, the Dofe group developed an ultrasound-assisted method for the synthesis of tetrazole-based isoxazoline derivatives **98** using a multi-step protocol. Starting from 1-(4-hydroxy-3-methoxyphenyl)ethenone **6** alkylation with chloroacetonitrile in DMF using K_2_CO_3_ yielded 1-(4-(cyanomethoxy)-3-methoxyphenyl)ethanone **97** (90% yield). Subsequent cyclization with sodium azide (NaN_3_) and ZnBr_2_ in H_2_O at 100 °C afforded the tetrazole precursor 1-(4-((1*H*-tetrazol-5-yl)methoxy)-3-methoxyphenyl)ethanone **97** (87% yield non-ultrasound step). The final yield of 5-((4-(4,5-dihydro-5-phenylisoxazol-3-yl)-2-methoxyphenoxy)methyl)-1*H*-tetrazole derivatives **98** was 67~98% yield, significantly outperforming conventional methods in both time reduction (hours to minutes) and yield improvement. MTT assays against A549, HepG2 (liver), and MCF-7 (breast) cancer cells revealed potent activity. The compound **98a** exhibited the strongest inhibition against A549, with IC_50_ = 0.78 µM, while **98b** and **98c** and showed significant tubulin polymerization inhibition, comparable to combretastatin A-4 (CA-4). Molecular docking confirmed binding at the colchicine site of tubulin, mimicking CA-4. This study highlights ultrasound-enhanced efficiency, broad anticancer activity, and the tubulin-targeting mechanism of tetrazole-isoxazoline hybrids, offering a robust platform for novel anticancer drug development (Scheme 40) [75].

**Scheme 41:**
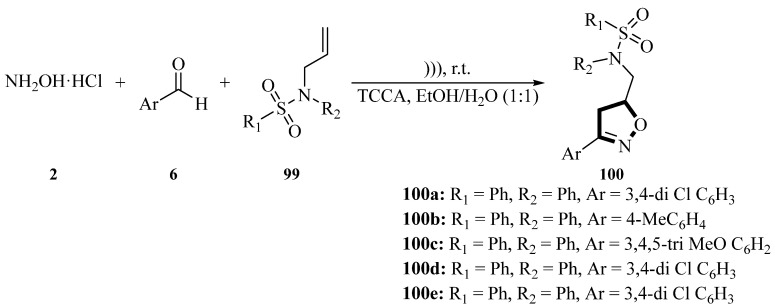
Synthesis of **100**.

In 2021, the Talha group published an article on a novel ultrasound-assisted one-pot three-component method for the synthesis of sulfonamide-isoxazoline hybrids **100** in which the target compounds **100** were obtained through the reaction of hydroxylamine hydrochloride **2**, aldehydes **6**, alkenes, and trichloroisocyanuric acid (TCCA). The study proposed a dual reaction mechanism supported by controlled experiments. Under ultrasonic irradiation, TCCA undergoes homolytic cleavage of the N–Cl bond to generate chlorine radicals and dichloroisocyanuric acid radicals, which oxidize aldoximes to unstable biradical nitrile oxides. These intermediates undergo regioselective 1,3-dipolar cycloaddition with alkenes to form 4-substituted isoxazolines **100**. Alternatively, TCCA may generate electrophilic chlorine atoms and cyanurate anions through mechanical effects, promoting an ionic pathway to nitrile oxides that similarly react with alkenes. The reaction was conducted in ethanol/water (1:1 *v*/*v*) under ultrasonic activation (80 W, 47 kHz) at 25 °C. TCCA served as both an oxidant and chlorinating agent, enabling aldehyde-to-nitrile oxide conversion without an additional base. Optimal conditions, using 0.5 mmol of TCCA, afforded products with 52~89% yields within 12~22 min. The synthesized sulfonamide-isoxazoline hybrids were evaluated for antineoplastic activity against hematological malignancies (K562 and HL-60 cell lines). Compound 3h demonstrated significant activity with an EC_50_ of 60~64 μM against HL-60 cells, inducing caspase-dependent apoptosis as evidenced by PARP and caspase-3 cleavage. Co-treatment with the pan-caspase inhibitor Q-VD-OPh fully restored cell viability, confirming the apoptotic mechanism. The marked difference in activity between **100a** and **100e** highlights the critical role of sulfonamide substituents in biological efficacy. This green, efficient methodology provides a valuable framework for developing novel antileukemic agents bearing sulfonamide-isoxazoline pharmacophores (Scheme 41) [76].

**Scheme 42:**
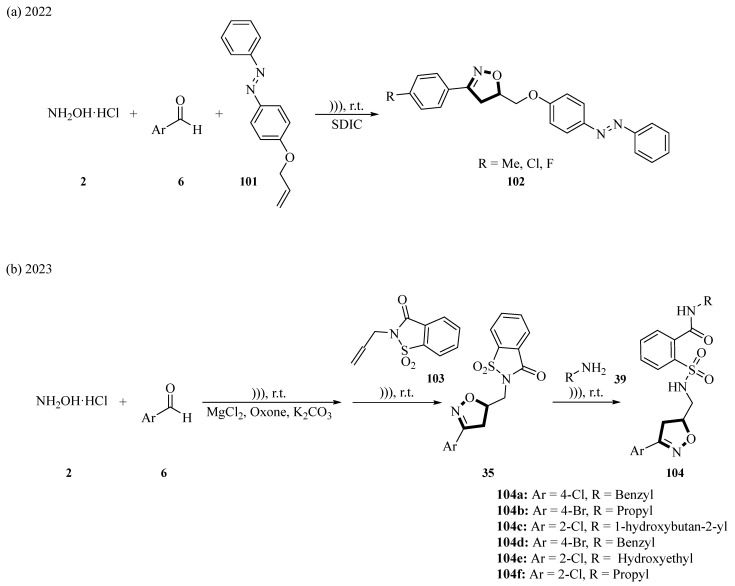
Synthesis of **102** and **104**.

In 2022, the Mahmoudi group developed a green, one-pot, three-component method for the synthesis of novel azo-isoxazolines **102** via ultrasound-assisted 1,3-dipolar cycloaddition. The reaction involved hydroxylamine hydrochloride **2**, aromatic aldehydes **6**, and 4-(allyloxy)azobenzene **101** as substrates. The process began with the in situ generation of nitrile oxides from hydroxylamine hydrochloride **2** and aldehydes **6** under sodium dichloroisocyanurate (SDIC)-mediated oxidation in H_2_O. These nitrile oxides then underwent regioselective 1,3-dipolar cycloaddition with 4-(allyloxy)azobenzene **101** under ultrasonic cavitation (47 kHz, 80 W) at room temperature, yielding 75~90% azo-isoxazolines **102** within 25~30 min. The method utilized H_2_O as a green solvent and SDIC as an eco-friendly, cost-effective oxidant, eliminating the need for toxic catalysts or organic solvents (Scheme 42a) [77].

The following year, Mahmoudi and co-workers successfully synthesized novel 3,5-disubstituted isoxazoline-sulfonamide derivatives **104** via a green ultrasound-assisted four-component reaction in the aqueous phase. The reaction involved hydroxylamine hydrochloride **2**, aromatic aldehydes **6**, pre-synthesized *N*-allyl-saccharin **35**, and primary amines as substrates. The reactions were consistent with the 2022 protocol in terms of temperature, ultrasound assistance, and solvent selection, with 70~95% yields achieved in 21~26 min, highlighting their efficiency and mild conditions. Bioassays against *Sphodroxia maroccana* larvae revealed exceptional larvicidal activity for halogenated derivatives **104a**~**104f**, with **104c** showing an LC_50_ of 0.18 mg/mL, outperforming fluralaner (LC_50_ = 0.99 mg/mL). Both studies used experiments to confirm that the reactions were both somewhat regioselective. The first study focused on the global significance of this research, which was primarily concerned with the application of green chemistry in the field of chemical synthesis, while the second study focused on solving the problem of agricultural pests in a specific region, particularly *Sphodroxia maroccana Ley*, a cork oak pest in Morocco (Scheme 42b) [78].

**Scheme 43:**
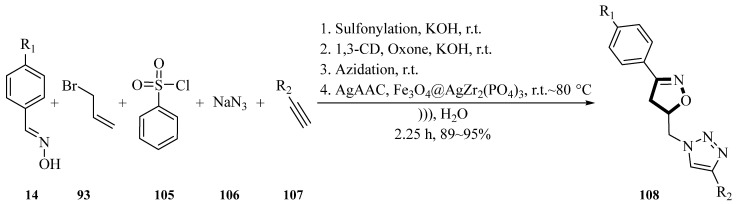
Synthesis of **108**.

In 2024, the Bougrin group cleverly synthesized isoxazolines **108** through a one-pot five-component four-step reaction by modifying the reaction products based on their research on ultrasound-assisted and Fe_3_O_4_@AgZr_2_(PO_4_)_3_ nanocatalyst synthesis of isoxazoles. This reaction employed benzenesulfonyl chloride **105** as the sulfonylation reagent and potassium hydroxide as the base, reacting with allyl alcohol in water at 25 °C, and combined aryl aldehyde oxime **14**, sodium azide **106**, and terminal alkynes **107** to synthesize isoxazoline compounds **108**. Their ultrasound-assisted aqueous system (20 kHz, 60% power) under temperature gradient conditions (25~80 °C) reduced the reaction time from 24 h to 75 min (total duration 135 min), with yields of product **108** reaching 89~95%. This method represents an advancement in atom-economical heterocyclic synthesis, addressing limitations such as high energy consumption and poor regioselectivity through innovative catalyst design and sonochemistry activation (Scheme 43) [47].

### 2.5. Synthesis of Isoxazolidine-Based Derivatives

**Scheme 44:**
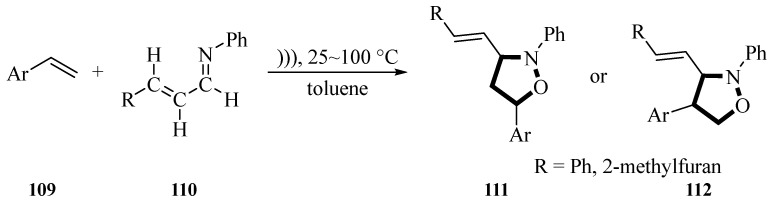
Synthesis of **111** or **112**.

In 1988, Borthakur and Sandhu utilized conjugated (*E*)-N-phenylbut-3-en-1-imine **110** and inactivated alkenes **109** as reactants. A Virsonic cell disruptor with an immersed sonic horn was employed as the ultrasound source, and the reaction mixture in toluene was sonicated for 5 min, followed by 5 min of standing. The experimental results demonstrated that the reaction time under ultrasound synthesis was significantly shorter compared with thermal conditions; for instance, the reaction of nitrile with styrene can save up to 33 h under ultrasonic conditions with a constant yield. The only isolable products in these experiments were isoxazolidines **111** or **112**, and the formation of regioisomer was explicitly ruled out. Ultrasound technology exhibited notable advantages in 1,3-dipolar cycloaddition reactions and provides a more efficient pathway for cycloaddition reactions in organic synthesis reveal the potential application of ultrasound in low-activity substrate reactions (Scheme 44) [31].

**Scheme 45:**
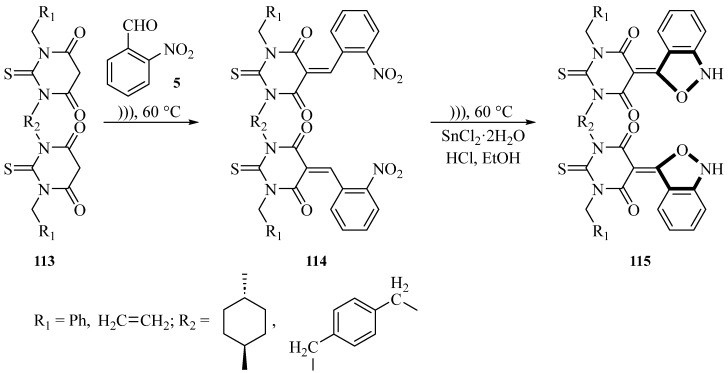
Synthesis of **115**.

In 2019, the Arafa group described the synthesis of bis-2,1-benzisoxazoles **115** possessing exocyclic nitrogen at the 5-position via reductive cyclization of bis-5-(2-nitrobenzylidene)thiobarbiturates **114**. The one-pot consecutive reaction utilized bis-thiobarbituric acid derivatives **113**, 2-nitrobenzaldehyde **5**, SnCl_2_·2H_2_O, and concentrated hydrochloric acid under ultrasound irradiation. Ultrasound irradiation significantly reduced reaction time and improved yields by about 30%. Regioselectivity correlated with the p*K*_a_ values of the parent amines: the amine with the higher p*K*_a_ participated in ring nitrogen formation, while the lower-p*K*_a_ amine remained as an exocyclic nitrogen in the triazole or thiazole moieties. This method demonstrated broad functional group tolerance and scalability (gram-scale synthesis), producing diverse derivatives such as bis-triazoles and bis-thiazolines with 89~99% yields. The protocol scored favorably in terms of green metrics (e.g., low *E*-factor, high atom economy), aligning with sustainable chemistry principles. Its efficiency, operational simplicity, and environmental compatibility highlight its significance in pharmaceutical and industrial applications (Scheme 45) [79].

## 3. Conclusions

With the intensive development of the green chemistry concept in recent years, ultrasound-assisted synthesis technology has shown great potential for the preparation of isoxazole and its derivatives due to its unique advantages. As an efficient and environmentally friendly synthesis method, ultrasound technology can significantly improve the reaction rate and product yield through the cavitation effect and reduce dependence on hazardous solvents and high temperature conditions.

In this study, we systematically summarized the recent advances in ultrasound-assisted isoxazole-based molecule synthesis, covering the optimization of classical reactions, the design of novel catalytic systems, and the construction of multifunctional isoxazole skeletons. We have shown that ultrasound irradiation not only simplifies the operation steps but also expands the range of substrate applicability, providing a new strategy for the efficient preparation of complex isoxazole derivatives. Recent innovations further highlight the versatility of ultrasound. For example, ionic liquids (ILs), such as polyoxometalate-based dicationic ionic liquid (POM-DIL), combined with sonication have enabled one-pot syntheses of substituted oxazoles with higher yield.

Although ultrasound-assisted synthesis has achieved remarkable results, some challenges still exist. For example, the sensitivity of some reactions to ultrasound parameters has not been fully clarified, the recycling efficiency of catalysts needs to be improved, and the stereoselective control of some complex isoxazole derivatives still needs to be explored. In addition, the synergistic effect of ultrasound technology with other emerging technologies (microwave-assisted, flow chemistry) has not been fully developed. Hybrid methodologies integrating ultrasound with microwave irradiation or flow chemistry are expanding the scope of accessible isoxazole architectures while enhancing scalability for industrial applications.

In conclusion, ultrasound-assisted synthesis technology has injected new vitality into isoxazole chemistry, and its high efficiency and environmental friendliness perfectly meet the development needs of sustainable chemistry. With the in-depth analysis of the reaction mechanism and the continuous innovation of technology integration, the ultrasonic irradiation strategy is expected to play a bigger role in the fields of agrochemical research, drug discovery, material science, and industrial catalysis, and provide solid support for the creation of functionalized isoxazole-based molecules.

## Data Availability

No new data were created or analyzed in this study. Data sharing is not applicable.
